# Stereotactic Body Radiation Therapy versus Concurrent Chemoradiotherapy for Locally Advanced Pancreatic Cancer: A Propensity Score-Matched Analysis

**DOI:** 10.3390/cancers14051166

**Published:** 2022-02-24

**Authors:** Young Seob Shin, Hee Hyun Park, Jin-hong Park, Dong-Wan Seo, Sang Soo Lee, Changhoon Yoo, Seonok Kim, Sang Min Yoon, Jinhong Jung, Myung-Hwan Kim, Sung Koo Lee, Do Hyun Park, Tae Jun Song, Dongwook Oh, Baek-Yeol Ryoo, Heung-Moon Chang, Kyu-pyo Kim, Jae Ho Jeong, Jong Hoon Kim

**Affiliations:** 1 Department of Radiation Oncology, Asan Medical Center, University of Ulsan College of Medicine, Seoul 05505, Korea; speed686@amc.seoul.kr (Y.S.S.); victoria41@naver.com (H.H.P.); drsmyoon@amc.seoul.kr (S.M.Y.); jung.jinhong@amc.seoul.kr (J.J.); jhkim2@amc.seoul.kr (J.H.K.); 2 Department of Gastroenterology, Asan Medical Center, University of Ulsan College of Medicine, Seoul 05505, Korea; dwseoamc@amc.seoul.kr (D.-W.S.); ssleedr@amc.seoul.kr (S.S.L.); mhkim@amc.seoul.kr (M.-H.K.); sklee@amc.seoul.kr (S.K.L.); dhpark@amc.seoul.kr (D.H.P.); medi01@naver.com (T.J.S.); dwoh@amc.seoul.kr (D.O.); 3 Department of Oncology, Asan Medical Center, University of Ulsan College of Medicine, Seoul 05505, Korea; yooc@amc.seoul.kr (C.Y.); ryooby@amc.seoul.kr (B.-Y.R.); changhm@amc.seoul.kr (H.-M.C.); kkp1122@amc.seoul.kr (K.-p.K.); jaeho.jeong@amc.seoul.kr (J.H.J.); 4 Department of Clinical Epidemiology and Biostatistics, Asan Medical Center, University of Ulsan College of Medicine, Seoul 05505, Korea; seonok@amc.seoul.kr

**Keywords:** pancreatic neoplasms, radiosurgery, chemoradiotherapy, treatment outcome

## Abstract

**Simple Summary:**

In the lack of direct comparative evidence of stereotactic body radiation therapy, we reviewed one of the largest locally advanced pancreatic cancer cohort homogeneously treated in a tertiary cancer center. Our propensity score–matched analysis shows comparable outcomes between stereotactic body radiation therapy and concurrent chemoradiotherapy in terms of survival, local control, and treatment-related toxicities. Considering the advantages of SBRT such as short treatment duration, better tolerance, easy combination with systemic treatment, and the potential for dose escalation, further investigation of the feasibility of SBRT as an alternative to CCRT in treating locally advanced pancreatic cancer is required.

**Abstract:**

In locally advanced pancreatic cancer (LAPC), stereotactic body radiation therapy (SBRT) has been applied as an alternative to concurrent chemoradiotherapy (CCRT); however, direct comparative evidence between these two modalities is scarce. The aim of this study was to compare the clinical outcomes of SBRT with CCRT for LAPC. We retrospectively reviewed the medical records of patients with LAPC who received SBRT (*n* = 95) or CCRT (*n* = 66) with a concurrent 5-FU-based regimen between January 2008 and July 2016. The clinical outcomes of freedom from local progression (FFLP), progression-free survival (PFS), overall survival (OS), and toxicities were analyzed before and after propensity score (PS) matching. After a median follow-up duration of 15.5 months (range, 2.3–64.5), the median OS, PFS, and FFLP of the unmatched patients were 17.3 months, 11 months, and 19.6 months, respectively. After PS matching, there were no significant differences between the SBRT and CCRT groups in terms of the 1-year rates of OS (66.7% vs. 80%, *p* = 0.455), PFS (40.0% vs. 54.2%, *p* = 0.123), and FFLP (77.2% and 87.1%, *p* = 0.691). Our results suggest SBRT could be a feasible alternative to CCRT in treating patients with LAPC.

## 1. Introduction

Pancreatic cancer is an aggressive malignancy, and only 10–20% of newly diagnosed patients are suitable for complete resection, which is considered the only curative approach [1]. Unresectable, or locally advanced pancreatic cancer (LAPC), accounts for about 30% of all pancreatic cancer, and although various combinations of chemotherapy and radiotherapy (RT) have been tried to improve the oncologic outcome, patients with LAPC still have a dismal prognosis with a median survival of 5–15 months [2,3]. The comparison between conventional concurrent chemoradiotherapy (CCRT) and chemotherapy alone showed contradictory results in several large LAPC trials, including the recent LAP07 study [4,5,6,7]. This phase III randomized study failed to show a significant gain in survival after CCRT despite better local control and similar toxicity rates [8]. The authors commented that further intensification of treatment strategies to treat early micrometastatic spread and enable downstaging might be necessary to achieve better oncologic outcomes.

Stereotactic body radiotherapy (SBRT) is a modern RT technique that has several advantages over conventional RT and has been widely applied as an effective local therapy for various types of cancers. SBRT enables the conformal and accurate delivery of high radiation doses in a few fractions while minimizing the irradiation of surrounding normal tissues. SBRT is considered to have different tumoricidal mechanisms, such as vascular endothelial destruction and immune modulation [9,10].

In LAPC, several studies reported favorable oncologic results of SBRT as an alternative treatment to CCRT [11,12,13,14,15]. However, there are few studies that compared SBRT with conventional CCRT or chemotherapy, and there is still a lack of empirical evidence for this new technique [16,17,18,19]. Therefore, in the present study, we compared the oncologic outcomes of patients with LAPC treated with SBRT or CCRT in order to provide detailed information about the optimal treatment strategy for LAPC.

## 2. Materials and Methods

### 2.1. Patients

We retrospectively reviewed the medical records of patients with histologically confirmed pancreatic cancer who were treated at our institution between January 2008 and July 2016. The eligibility criteria were as follows: (1) unresectable tumor classified by the multidisciplinary oncology team review of imaging studies according to the National Comprehensive Cancer Network (NCCN) guideline [20]; (2) underwent SBRT or CCRT; (3) no distant metastases at baseline or before RT; (4) Eastern Cooperative Oncology Group (ECOG) performance status of 0 to 2. The initial patient evaluation included physical examination, complete blood count, standard blood chemistry panel including carbohydrate antigen 19-9 (CA19-9), pancreatic protocol computed tomography (CT) scan, chest radiograph, magnetic resonance imaging (MRI), and positron emission tomography-CT (PET-CT) scan. This study was approved by the institutional review board of Asan Medical Center, and informed consent was waived due to the retrospective nature of the study.

### 2.2. Treatment

The use of induction chemotherapy and the method of RT were determined at the discretion of treating physicians. Induction chemotherapy was defined as the start of chemotherapy more than 1 month prior to RT. For SBRT, the respiratory-gated intensity-modulated radiation therapy (IMRT) or volumetric modulated arc radiotherapy (VMAT) technique was used. The SBRT procedure used at our institution was described in our previous papers [21,22]. Briefly, a four-dimensional CT (GE LightSpeed RT 16; GE Healthcare, Waukesha, WI, USA) simulation was performed during free breathing. A Real-time Position Management Respiratory Gating system (Varian Medical Systems, Palo Alto, CA, USA) was used to record the patients’ breathing patterns. The CT data were sorted according to the respiratory phase, and treatment planning was performed based on the CT images at the end-expiratory phase.

Both the primary tumor and metastatic regional lymph nodes were included in the gross tumor volume (GTV) when target coverage and dose constraints could be maintained. A lymph node was regarded as metastasis if it was more than 1 cm in short-axis diameter or if it had necrotic features. Diagnostic CT, MRI, and PET-CT images were used to assist in defining the GTV. To reduce internal motion margins, a respiratory gating scheme around the end-expiratory phase (30 to 70% in most cases) was applied to all patients. The maximum intensity projection (MIP) images corresponding to the gating window were consulted to contour the internal target volume (ITV). Three or four gold seeds implanted near the primary tumor or pancreatic duct stent were used as an internal marker, and full-phase trajectory was delineated. The planning target volume (PTV) was defined using 3 mm isotropic margins to the ITV in order to account for set-up errors, unless the margin resulted in expansion into the duodenum or stomach; in such cases, a non-uniform PTV margin expansion was used provided that the GTV dose constraints were met. The prescribed dose was administered to the isodose line covering the PTV.

The total dose was mainly determined based on general dosing guidelines after determining the dose to be administered to the normal organs, including the following: maximal point dose to the stomach, duodenum, or small bowel was kept to <30 Gy, and ≥700 cm^3^ of the normal liver was kept to <15 Gy. The volume of 75% of combined kidneys was kept to <12 Gy, and the maximal point dose to the spinal cord was <20 Gy.

All patients in the CCRT group were treated with three-dimensional conformal RT (3DCRT). In addition to the primary tumor and metastatic lymph nodes, the inclusion of regional lymphatics in the clinical target volume was decided by the physician based on the patient’s performance and disease status. A PTV margin of 0.7–1.0 cm was added for daily set-up variations. During RT, all patients received concurrent oral capecitabine or intravenous 5-fluorouracil (5-FU) bolus with leucovorin.

### 2.3. Follow-Up and Toxicity Evaluation

After treatment, regular follow-up examinations were performed at 2 to 3-month intervals. Follow-up evaluation included physical examination, complete blood count, standard blood chemistry panel including CA19-9, and an abdominal CT scan. Additional imaging studies were conducted whenever clinically indicated. A contrast-enhanced abdominal CT scan was used for the assessment of treatment response. For patients who responded sufficiently, decision to proceed with surgical resection was made by the multidisciplinary team. Local failure was defined as growth of the radiated pancreatic lesion or regional lymph nodes, and distant failure was defined as clinical or pathological detection of disease beyond the pancreas and regional lymph nodes. During and after treatment, treatment-related toxicities were reported using the Common Terminology Criteria for Adverse Events, version 4.0 (https://ctep.cancer.gov/protocoldevelopment/electronic_applications/ctc.htm, accessed on 23 February 2022). Events reported within 90 days after RT were classified as acute toxicities, whereas those occurring after 90 days were considered late toxicities.

### 2.4. Statistical Analysis

Patient characteristics between the two treatment groups were compared by Student’s *t*-test for continuous variables and the χ2 test or Fisher’s exact test for categorical variables. The Kaplan–Meier method was used to estimate the rates of freedom from local progression (FFLP), progression-free survival (PFS), and overall survival (OS). FFLP was calculated from the date of diagnosis to the date of local failure by radiologic or pathologic examination. PFS was calculated from the date of diagnosis to the date of any progression or death from any cause. OS was calculated from the date of diagnosis to the date of death from any cause. Comparison of survival rates was performed with the log-rank test. Cox proportional hazards model was used to assess the level of statistical significance of prognostic factors for OS. Multivariable analysis was performed with backward elimination of all variables with a *p* value of <0.2 in the univariate analysis. The cumulative incidence of local recurrence (LR) was estimated and compared using Gary’s test considering death as a competing risk. To identify the risk factors, we used Fine and Gray’s method for modeling the hazard of the sub-distribution to account for death as a competing risk [23].

Propensity scores were generated using the logistic regression model that included age, sex, performance status, LN metastasis, tumor abutment to stomach and duodenum, location, size, and pre-RT CA19-9. Greedy matching was performed using a caliper of 0.2 standard deviations of the logit of the propensity score. The absolute standardized differences were used to diagnose the balance after propensity score matching, and all absolute standardized differences were less than 0.15 after matching. All analyses were performed using SAS 9.4 (SAS Institute, Cary, NC, USA) and SPSS 22.0 (IBM, Armonk, NY, USA).

## 3. Results

### 3.1. Baseline Characteristics and Treatment

Patient characteristics at the time of radiotherapy are summarized in Table 1. Of the 161 patients, 95 and 66 patients underwent SBRT and CCRT, respectively; 93 (57.8%) patients had a pancreatic head tumor and 114 (70.8%) had clinical N0 disease. Compared with the CCRT group, the SBRT group had a significantly older age (median, 64.0 vs. 60.5, *p* = 0.045) and higher proportions of patients with tumors larger than 40 mm (66.3% vs. 24.2%, *p* < 0.001) and CA 19-9 levels higher than 37 U/mL (79.9% vs. 63.6%, *p* = 0.032). Induction chemotherapy was administered to 40 (42.1%) patients in the SBRT group and 60 (90.9%) patients in the CCRT group (*p* < 0.001); of the patients who received induction chemotherapy, 32 (32%) received FOLFIRINOX (5-FU, leucovorin, oxaliplatin, and irinotecan) or gemcitabine/nanoparticle albumin-bound (nab)-paclitaxel, while others received other gemcitabine-based regimens. In the SBRT group, 92 (94.7%) patients received 4-fraction (Fx) treatment and the median dose was 28 Gy (range, 24–36). The median dose in the CCRT group was 54 Gy (range, 40–59.4) in 1.8- or 2.0-Gy per Fx. Except for three patients (one with 40 Gy and two with 46 Gy), all patients were treated with doses higher than 50 Gy. Additional chemotherapy was administered to 82 (86.3%) patients in the SBRT group and 16 (24.2%) patients in the CCRT group (*p* < 0.001). Post-RT chemotherapy regimens were FOLFIRINOX or gemcitabine/nab-paclitaxel in seven patients and other gemcitabine-based regimens in 87 patients.

### 3.2. Survival Outcomes

**Figure 1:**
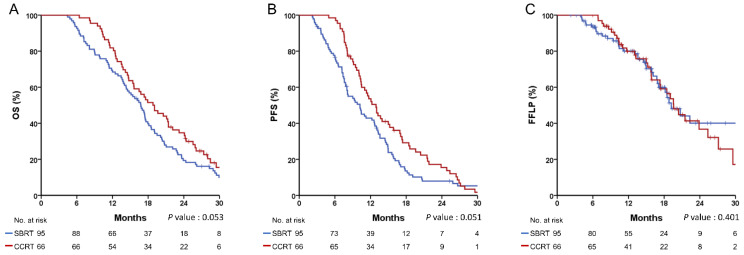
Recurrence and survival outcomes in the unmatched cohort. (**A**) overall survival (OS); (**B**) progression-free survival (PFS); (**C**) freedom from local progression (FFLP).

The median follow-up duration was 15.5 months (range, 2.3–64.5), and the median OS, PFS, and FFLP of the total patients were 17.3 months, 11 months, and 19.6 months, respectively. In the SBRT and CCRT groups, the 1-year rates of OS, PFS, and FFLP were 68.4% and 81.8% (*p* = 0.053, Figure 1A), 42.9% and 53.6% (*p* = 0.051, Figure 1B), and 80.4% and 80.0% (*p* = 0.401, Figure 1C), respectively. Multivariate analysis showed that tumor size > 40 mm (hazard ratio (HR), 1.463; 95% confidence interval (CI), 1.034–2.070), curative resection (HR, 0.324; 95% CI, 0.149–0.704), and induction chemotherapy duration > 6 months (HR, 0.572; 95% CI, 0.341–0.958) were significantly associated with OS (Table 2). There were no significant predictive factors for LR in the multivariate analysis (Table 3), and the type of RT was not significantly associated with the OS or LR.

### 3.3. Propensity Score-Matched Analysis for Survival Outcomes

**Figure 2:**
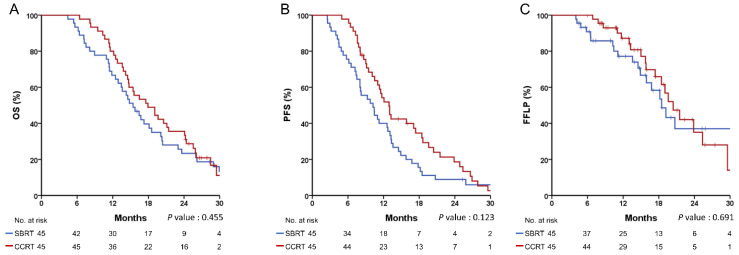
Recurrence and survival outcomes in the propensity score-matched cohort. (**A**) overall survival (OS), (**B**) progression-free survival (PFS), (**C**) freedom from local progression (FFLP).

By using the propensity score, 45 patients in each group were matched. The baseline characteristics were well balanced between the matched groups, except for the sequence of chemotherapy (Table 4). There were no significant differences between the two groups in the 1-year rates of OS (66.7% vs. 80.0%, *p* = 0.455, Figure 2A), PFS (40.0% vs. 54.2%, *p* = 0.123, Figure 2B), and FFLP (77.2% vs. 87.1%, *p* = 0.691, Figure 2C). The cumulative incidence of LR at 1 year was not significantly different after considering death as a competing risk (22.4% vs. 12.9%, *p* = 0.906).

### 3.4. Events after Treatment

**Figure 3:**
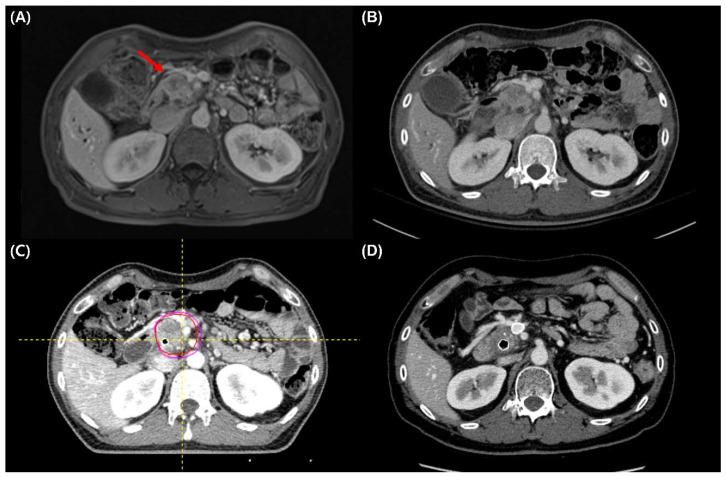
A representative patient who underwent curative resection after stereotactic body radiation therapy (SBRT). (**A**,**B**) Pre-SBRT magnetic resonance imaging and computed tomography (CT) show pancreatic head cancer (red arrow) abutting more than 180° of the superior mesenteric artery (SMA). (**C**) Gross tumor volume and planning target volume in simulation CT. (**D**) Three months follow-up CT after SBRT shows decreased tumor and SMA abutment.

During a median duration of 3.2 months (range, 2.7–5.6) after the end of RT, seven (7%) patients in the SBRT group and two (3%) patients in the CCRT group underwent curative resection (*p* = 0.311; Table 5). Images of a representative patient who underwent curative resection after SBRT are presented in Figure 3. The median OS of these nine patients was 28 months (range, 16–65), and R0 resection was achieved in six patients. After surgery, three patients showed distant recurrence, and another three patients showed simultaneous local and distant recurrence. Two (one in each group) patients were still alive at the last follow-up without recurrence. During the follow-up period, 79 (83.2%) patients in the SBRT group and 56 (84.8%) patients in the CCRT group experienced disease progression or recurrence, and the first sites of failure were predominantly distant in both groups (Table 5). Twelve patients (12.6%) in the SBRT group and 17 patients (25.8%) in the CCRT group had an isolated local recurrence (*p* = 0.102).

### 3.5. Toxicities

There were no significant differences between the two groups in the occurrence of acute and late toxicities (Table 5). For acute toxicities, one patient in the SBRT group had nausea and abdominal pain 2 months after RT, and abdominal CT showed peritoneal-free air, suggesting gastric ulcer perforation; the patient was managed with conservative care, and peritoneal air was not evident in the follow-up CT. One patient in the CCRT group stopped the treatment at 50.4 Gy due to duodenal ulcer bleeding at the tumor infiltration site. Bleeding was controlled with conservative treatment, but the patient died of peritoneal tumor progression after 2 months. Another patient in the CCRT group presented with abdominal pain 2 months after treatment, and the abdominal CT showed sealed duodenal perforation. Because the perforation occurred near the duodenal stent that had been inserted before treatment, it was not considered to be related to treatment and was managed conservatively. Other cases of acute toxicities such as nausea and anemia all improved soon after the treatment.

For late toxicities, duodenal ulcer bleeding occurred in two patients in the SBRT group and one patient in the CCRT group at 4–6 months after RT; because, the tumors were attached to or invading the ulcer site, it was difficult to determine the causal relationship between treatment and ulcer. Likely owing to their tumor-related nature, these lesions waxed and waned during follow-up. No patients died due to treatment-related toxicity.

## 4. Discussion

RT has been widely used for treating LAPCs and has been shown to be effective in achieving local control while preventing pain and obstructive symptoms that deteriorate the quality of life. However, regarding survival benefit, the role of conventional RT has shown contradictory results, and SBRT has been investigated as a potential alternative. In the present study, we reviewed one of the largest LAPC cohorts homogeneously treated in a tertiary cancer center and performed a comparison between SBRT and CCRT. The median survival duration of 17.3 months and the 1-year local control rate of 70–80% in our study were comparable to the previous results of phase III trials and meta-analyses [4,6,24]. After PS matching, there were no significant differences in the rates of survival, local control, and treatment-related toxicities between SBRT and CCRT.

Theoretically, SBRT could provide a greater local tumor control and lower toxicities through precise treatment delivery; accordingly, several studies have reported favorable oncologic outcomes of SBRT in LAPC [12,13,14,25,26]. However, high-quality evidence comparing CCRT and SBRT is still lacking. Park et al. compared unmatched 44 SBRT and 226 CCRT patients and reported similar disease control rates, in which the 1-year rates of OS and local failure were 56.2% vs. 59.6% (*p* = 0.75) and 34.4% vs. 30.2% (*p* = 0.51) in the two groups, respectively [17]. Lin et al. reported a small study comparing 20 SBRT and 21 IMRT patients, in which there was no significant difference in the 1-year OS (80.0% vs. 70.7%, *p* = 0.127), while SBRT was associated with better local control in multivariate analysis [16]. A recent Italian multicenter case-control study compared 40 matched pairs of SBRT and CCRT patients and showed that the SBRT group had a non-inferior OS (1 year, 79.8% vs. 73.8%, *p* = 0.470) but superior local control (1 year, 80.4% vs. 53.1%, *p* = 0.017) to the CCRT group [27]. In addition, a few meta-analyses and registry studies using the National Cancer Data Base reported that SBRT was associated with improved overall survival compared with CCRT [18,19,28].

Regarding treatment-related toxicities, the treatments were well-tolerated in both groups, and there were no significant differences in the incidence of severe (≥grade 3) acute and late toxicities. An Italian study similarly reported that there were no significant differences in the acute and late gastrointestinal toxicities between the SBRT and CCRT groups [27]. However, in both studies, the rate of acute toxicity was higher in the CCRT group, albeit without statistical significance. On the other hand, Park et al. [17] showed a significantly higher rate of acute grade 2 gastrointestinal toxicity in the CCRT group compared with the SBRT group (24% vs. 7%, *p* = 0.008). Similarly, in a meta-analysis of 9 SBRT and 11 CCRT investigations, severe acute toxicities were more prevalent in the CCRT group than in the SBRT group (37.7% vs. 5.6%, *p* = 0.0002), while there was no significant difference in severe late toxicities (10.1% vs. 5.9%, *p* = 0.49).

In the absence of a randomized trial, it is difficult to conduct a fair comparison between SBRT and CCRT. Many confounders, such as disease extent, tumor location, induction chemotherapy, socioeconomic status, and type of treatment center, could influence the choice of treatment method. Our propensity score-matched study provides the same result as previous reports that SBRT leads to non-inferior outcomes compared with CCRT. However, unlike several other previous studies, we did not observe significantly superior results in the SBRT group in terms of local control, survival, or toxicity. The reason for the lack of a significant difference might be that our cohort size was not sufficiently large or that confounding variables such as tumor location and tumor-bowel abutment were more well-controlled than in other studies. Further investigation is required to determine whether SBRT could provide improved local control, survival, or toxicity compared with CCRT.

SBRT is considered to have additional advantages in the current treatment strategy. The failure of several previous randomized trials in demonstrating the survival benefits of RT was partially due to the fact that distant metastasis was the dominant primary pattern of failure in LAPC patients [4,6,18]. In the present study, the first site of recurrence was a distant location in more than 60% of patients. In such patients, improvements in local control are unlikely to significantly improve their survival. However, recently applied intensified chemotherapy regimens such as FOLFIRINOX and gemcitabine/nab-paclitaxel have demonstrated significantly improved response rates and longer survival in pancreatic cancer [29,30,31,32,33,34]. As systemic control improves, local control can play a more critical role in the patients’ oncologic outcomes; therefore, the roles of RT and RT methods need to be re-evaluated. In this regard, SBRT would be more advantageous than CCRT as it minimizes chemotherapy interruption with short treatment duration and better patient tolerance. Most patients in our SBRT group started post-SBRT chemotherapy within 2 weeks, and previous studies also reported the feasibility of chemotherapy resuming 1 week after SBRT [16,25,26,35].

As several studies have demonstrated the dose-response relationship for RT in pancreatic cancer [24,36], a higher dose of RT is desirable for better tumor control. However, administering high-dose RT to the pancreas is difficult as it is surrounded by radiosensitive organs such as the stomach and duodenum. Several different dose and fractionation schemes have been attempted, and some of them that used single Fx were associated with intolerable severe GI toxicities [11,14]. Accordingly, multi-fraction treatment has been widely used in recent and ongoing trials as well as ours [37]. These multi-fraction regimens are currently considered feasible in terms of efficacy and toxicity; however, further dose escalation is suggested when using SBRT with recently developed RT techniques such as MR-guided adaptive RT or simultaneous integrated boost [38,39,40]. Another potential benefit of dose escalation is converting an unresectable LAPC to a resectable one. In the present study, approximately 7% of patients received curative resection after SBRT, which is in line with several previous reports [8]. However, surgical conversion may be a critical issue in future investigations considering that a much higher conversion rate is expected with an intensified chemotherapy regimen [33,41]. To improve the resectability by reducing the tumors around crucial vessels, several ongoing trials prescribe higher doses whenever possible to tumor-vessel interfaces with advanced RT techniques [39,40]. The results of these new RT techniques and higher doses should be followed up.

Our study has several limitations. First, the retrospective nature of this study confers potential selection biases. At our institution, there were no policies to skew certain patients towards treatment; however, the selection between SBRT and CCRT was not randomized. Although we conducted propensity score matching to reduce selection biases, several factors, such as number and schedule of chemotherapy, were not included in the propensity score generation and biases could not be completely eliminated. Second, our cohort included treatment-naïve patients as well as those who had received different numbers of induction chemotherapy. Given that we evaluated variables at the time of RT initiation, induction chemotherapy could have affected baseline characteristics and survival estimations. However, in clinical practice, patients who show a good response after induction chemotherapy receive RT, and they usually have a better prognosis than treatment-naïve patients. In the present study, this bias might have worked in favor of the CCRT group, which had a higher proportion of patients who had completed induction chemotherapy than the SBRT group. Therefore, we believe that this bias actually supports the non-inferiority of SBRT. Third, because the majority of the patients were treated before the introduction of FOLFIRINOX or gemcitabine/nab-paclitaxel, these patients did not receive the best treatment options by the current standard. Although our study focuses on RT rather than chemotherapy, the comparison of RT methods with intensified regimens should be investigated in the future. Despite these limitations, to our knowledge, the present study is one of the largest matched studies reporting the non-inferiority of the current fractionated SBRT scheme to CCRT. Further investigations are warranted to confirm our results.

## 5. Conclusions

In the current study, SBRT was not inferior to CCRT for patients with LAPC in terms of local control, survival, and toxicity. Considering the advantages of SBRT, such as short treatment duration, better tolerance, easy combination with systemic treatment, and the potential for dose escalation, further investigation of the feasibility of SBRT as an alternative to CCRT is required.

## Figures and Tables

**Table 1:** Characteristics of the unmatched cohort at the time of radiotherapy.

Variable	SBRT (*n* = 95)	CCRT (*n* = 66)	*p* value	ASD
Age (years)	64 (38–84)	60.5 (34–76)	0.045	0.346
Gender			0.057	0.320
Male	49 (51.6%)	44 (66.7%)	-	-
Female	46 (48.4%)	22 (33.3%)	-	-
ECOG PS			0.473	0.192
0–1	89 (93.7%)	64 (97.0%)	-	-
2	6 (6.3%)	2 (3.0%)	-	-
Pre-RT CA19-9			0.032	0.318
≤37 U/mL	20 (21.1%)	24 (36.4%)	-	-
>37 U/mL	75 (79.9%)	42 (63.6%)	-	-
Tumor Location			0.351	0.152
Head	52 (54.7%)	41 (62.1%)	-	-
Body/Tail	43 (45.3%)	25 (37.9%)	-	-
Tumor Size			<0.001	0.982
≤40 mm	32 (33.7%)	50 (75.8%)	-	-
>40 mm	63 (66.3%)	16 (24.2%)	-	-
Clinical Node Stage			0.095	0.253
N0	72 (75.8%)	42 (63.6%)	-	-
N+	23 (24.2%)	24 (36.4%)	-	-
Abutting the Stomach/Duodenum			0.515	0.101
No	72 (75.8%)	47 (71.2%)	-	-
Yes	23 (24.2%)	19 (28.8%)	-	-
Induction Chemotherapy			<0.001	-
None	55 (57.9%)	6 (9.1%)	-	-
≤6 months	38 (40.0%)	43 (65.2%)	-	-
>6 months	2 (2.1%)	17 (25.7%)	-	-
Post-RT Chemotherapy			<0.001	-
No	13 (13.7%)	50 (75.8%)	-	-
Yes	82 (86.3%)	16 (24.2%)	-	-
RT dose (EQD2, α/β = 10)	39.7 (31.3–57.0)	54.0 (40.0–58.4)	<0.001	-

Abbreviations: SBRT, stereotactic body radiotherapy; CCRT, concurrent chemoradiotherapy; ASD, absolute standardized difference; ECOG PS, Eastern Cooperative Oncology Group performance status; RT, radiotherapy; CA19-9, carbohydrate antigen 19-9; EQD2, equivalent dose in 2 Gy per fraction. Values are presented as median (range) or number (%) of patients.

**Table 2:** Variables associated with overall survival.

Variable	Univariate		Multivariate	
	*p* Value	HR (95% CI)	*p* Value	HR (95% CI)
Group	0.054	0.722	-	-
(SBRT vs. CCRT)		(0.518–1.006)		
Age	0.033	1.019	-	-
		(1.001–1.036)		
Gender	0.739	0.946	-	-
(M vs. F)		(0.681–1.313)		
ECOG PS	0.038	2.146	-	-
(0–1 vs. 2)		(1.044–4.411)		
Pre-RT CA19-9	0.074	1.400	0.056	1.452
(≤37 vs. >37)		(0.968–2.024)		(0.990–2.128)
Tumor location	0.165	0.791	-	-
(Head vs. Body/tail)		(0.568–1.102)		
Tumor size	0.005	1.625	0.032	1.463
(≤40 vs. >40)		(1.162–2.273)		(1.034–2.070)
Clinical N stage	0.524	1.125	-	-
(N0 vs. N+)		(0.783–1.618)		
Abutting the stomach/duodenum	0.052	1.438	-	-
(No vs. Yes)		(0.997–2.074)		
Induction CTx	0.365	0.858	-	-
(No vs. Yes)		(0.617–1.195)		
Induction CTx duration	0.017	0.540	0.034	0.572
(≤6 months vs. >6 months		(0.325–0.896)		(0.341–0.958)
RT dose (EQD2)	0.202	0.986	-	-
		(0.966–1.007)	-	-
Resection	0.022	0.410	0.004	0.230
(No vs. Yes)		(0.191–0.880)		(0.097–0.543)

Abbreviations: SBRT, stereotactic body radiotherapy; CCRT, concurrent chemoradiotherapy; ECOG PS, Eastern Cooperative Oncology Group performance status; RT, radiotherapy; CA19-9, carbohydrate antigen 19-9; CTx, chemotherapy; EQD2, equivalent dose in 2 Gy per fraction.

**Table 3:** Variables associated with local recurrence.

Variable	Univariate		Multivariate	
	*p* Value	HR (95% CI)	*p* Value	HR (95% CI)
Group	0.187	1.376	-	-
(SBRT vs. CCRT)		(0.847–2.212)		
Age	0.803	0.997	-	-
		(0.976–1.019)		
Gender	0.038	0.585	-	-
(M vs. F)		(0.353–0.972)		
ECOG PS	0.622	1.281	-	-
(0–1 vs. 2)		(0.479–3.430)		
Pre-RT CA19-9	0.022	0.564	0.080	0.631
(≤140 vs. >140)		(0.345–0.920)		(0.377–1.057)
Tumor location	0.209	0.733	-	-
(Head vs. Body/tail)		(0.451–1.191)		
Tumor size	0.021	0.558	0.073	0.623
(≤40 vs. >40)		(0.341–0.915)		(0.371–1.046)
Clinical N stage	0.204	1.399	-	-
(N0 vs. N+)		(0.834–2.346)		
Abutting the stomach/duodenum	0.882	1.044	-	-
(No vs. Yes)		(0.588–1.854)		
Induction CTx	0.796	0.937	-	-
(No vs. Yes)		(0.574–1.530)		
Induction CTx duration	0.762	0.929	-	-
(≤6 months vs. >6 months		(0.575–1.500)		-
RT dose (EQD2)	0.542	1.010	-	-
		(0.978–1.044)	-	-
Resection	0.971	0.983	-	-
(No vs. Yes)		(0.395–2.445)		-

Abbreviations: SBRT, stereotactic body radiotherapy; CCRT, concurrent chemoradiotherapy; ECOG PS, Eastern Cooperative Oncology Group performance status; RT, radiotherapy; CA19-9, carbohydrate antigen 19-9; CTx, chemotherapy; EQD2, equivalent dose in 2 Gy per fraction.

**Table 4:** Characteristics of the propensity score-matched cohort at the time of radiotherapy.

Variable	SBRT (*n* = 45)	CCRT (*n* = 45)	*p* Value	ASD
Age (years)	61 (39–84)	61 (37–76)	0.790	-
Gender			0.664	0.093
Male	27 (60.0%)	29 (64.4%)	-	-
Female	18 (40.0%)	16 (35.6%)	-	-
ECOG PS			1.000	0.000
0–1	43 (95.6%)	43 (95.6%)	-	-
2	2 (4.4%)	2 (4.4%)	-	-
Pre-RT CA19-9			0.655	0.093
≤37 U/mL	14 (31.1%)	16 (35.6%)	-	-
>37 U/mL	31 (68.9%)	29 (64.4%)	-	-
Tumor Location			1.000	0.000
Head	29 (64.4%)	29 (64.4%)	-	-
Body/Tail	16 (35.6%)	16 (35.6%)	-	-
Tumor Size, Median			0.827	0.046
≤40 mm	28 (62.2%)	29 (64.4%)	-	-
>40 mm	17 (37.8%)	16 (35.6%)	-	-
Clinical Node Stage			1.000	0.000
N0	32 (71.1%)	32 (71.1%)	-	-
N+	13 (28.9%)	13 (28.9%)	-	-
Abutting the Stomach/Duodenum			0.814	0.050
No	32 (71.1%)	33 (73.3%)	-	-
Yes	13 (28.9%)	12 (26.7%)	-	-
Induction Chemotherapy			<0.001	-
None	25 (55.6%)	2 (4.4%)	-	-
≤6 months	18 (40.0%)	26 (57.8%)	-	-
>6 months	2 (4.4%)	17 (37.8%)	-	-
Post-RT Chemotherapy			<0.001	-
No	6 (13.3%)	33 (73.3%)	-	-
Yes	39 (86.7%)	12 (26.7%)	-	-
RT dose (EQD2, α/β = 10)	39.7 (31.3–57.0)	53.1 (40.0–58.4)	<0.001	-

Abbreviations: SBRT, stereotactic body radiotherapy; CCRT, concurrent chemoradiotherapy; ASD, absolute standardized difference; ECOG PS, Eastern Cooperative Oncology Group performance status; RT, radiotherapy; CA19-9, carbohydrate antigen 19-9; EQD2, equivalent dose in 2 Gy per fraction. Values are presented as median (range) or number (%) of patients.

**Table 5:** Events after treatment.

Event	SBRT (*n* = 95)	CCRT (*n* = 66)	*p* Value
Curative resection	7 (7.4%)	2 (3.0%)	0.311
* Treatment-related toxicity (≥Grade 3)			
Acute	3 (3.2%)	5 (7.6%)	0.274
Late	2 (2.1%)	1 (1.5%)	1.000
First site of treatment failure	79 (83.2%)	56 (84.8%)	0.102
Local	12 (12.6%)	17 (25.8%)	-
Distant	55 (57.9%)	31 (47.0%)	-
Both	12 (12.6%)	8 (12.1%)	-

Abbreviations: SBRT, stereotactic body radiotherapy; CCRT, concurrent chemoradiotherapy. * Toxicity was assessed using Common Terminology Criteria for Adverse Events, version 4.0 (https://ctep.cancer.gov/protocoldevelopment/electronic_applications/ctc.htm, accessed on 23 February 2022). Events reported within 90 days after RT were classified as acute toxicities, whereas those occurring after 90 days were considered as late toxicities.

## Data Availability

The datasets used and/or analyzed during this study are available from the corresponding author upon reasonable request.
